# The contribution of amphibian macrophage subsets to scarless regeneration of skin wounds

**DOI:** 10.3389/fimmu.2025.1713361

**Published:** 2025-12-11

**Authors:** Christina N. Garvey Griffith, Kelsey A. Hauser, Bradley Abramson, Elissa R. Chapkin, Elizabeth J. Jones, Anju N. Duttargi, Leon Grayfer

**Affiliations:** 1 Department of Biological Sciences, George Washington University, Washington, WA, United States; 2 Noblis, Science Technology and Engineering, Reston, VA, United States; 3 Histopathology and Tissue Shared Resource, Lombardi Comprehensive Cancer Center, Georgetown University, Washington, WA, United States

**Keywords:** wound repair, regeneration, macrophage, interleukin-34, colony stimulating factor-1

## Abstract

*Xenopus laevis* juvenile frogs regenerate wounded skin without scarring, yet the underlying mechanisms driving this process remain poorly defined. Macrophages are critical to wound repair across vertebrates, and our results indicate a transient influx of macrophages into regenerating frog wounds. The colony stimulating factor-1 (CSF1) and interleukin-34 (IL34) growth factors control macrophage development. Through RNA *in situ* hybridization studies, we found that *csf1* gene expression peaked early during juvenile frog wound responses, whereas *il34* expression increased later in the repair process. Our past studies indicate that *X. laevis* CSF1- and IL34-differentiated macrophages are functionally distinct. Presently, we treated frog wounds with recombinant (r)CSF1 and rIL34 to determine the roles of the corresponding macrophage subsets in wound repair. Using a combination of RNA *in situ* hybridization, RNA sequencing and histology, we demonstrated that wounds skewed towards greater proportions of rCSF1-macrophages exhibited greater infiltration of leukocytes, chiefly amongst them neutrophils. These wounds also possessed robust expression of inflammatory genes and transcripts associated with granulation and fibrosis. By contrast, rIL34-treated frog wounds exhibited greater fibroblast activation concurrent with greater type I/III collagen ratios and expression of genes typically seen at later phases of wound repair. Together, we propose that while CSF1-macrophages are likely more prominently involved in the inflammatory phase of *X. laevis* wound repair, IL34-macrophages predominate the later reparative phase of these responses.

## 1 Introduction

Mammalian skin is composed of epidermal and dermal layers, and when the latter is damaged, the lost tissue is replaced with collagenous scar tissue, devoid of secondary structures such as sweat glands or hair follicles (1–3). These scars may be disfiguring and lack some of the functions of the original dermal tissue, often resulting in cosmetic and/or medical issues (4). Mammalian immune systems play critical roles during all phases of wound repair, from the initial inflammation to the resolution of the inflammatory response and the ensuing coordinated repair pathways (5). Notably, amphibians possess immune systems remarkably similar to those of mammals (6) and yet have impressive capacities to repair and regrow damaged tissues and even limbs (7, 8). Urodele amphibians (newts and salamanders), and pre-metamorphic anuran amphibians (frogs and toads) can regrow whole limbs and some organs. In contrast, post-metamorphic anurans lack these regenerative capacities but do exhibit a remarkable propensity to regenerate wounded skin without forming scars (4, 9, 10).

Macrophage (Mϕ) lineage cells are critical to skin wound repair (11, 12) including coordinating inflammation (4), clearing of debris and dead cells (13), promoting vascularization (4) and re-epithelialization (4), facilitating extracellular matrix deposition (14), and recruiting and activating fibroblasts (2, 15). The involvement of Mϕs in the process of wound repair appears to be evolutionarily conserved across vertebrates, from fish to mammals (5, 16–19). In the early phases of mammalian wound healing, resident and infiltrating Mϕs predominantly express pro-inflammatory mediators, transitioning to anti-inflammatory profiles as wound healing progresses (16). How Mϕ subtypes participate in wound repair outside of mammals remains largely unknown.

While vertebrates possess numerous Mϕ-lineage subsets (20, 21), the differentiation and functionality of all vertebrate Mϕs depend on the colony-stimulating factor-1 receptor (CSF1R), which is ligated by CSF1 and interleukin-34 (IL34) cytokines (22). Dimeric IL34 and CSF1 both interact with dimerized CSF1R (23), with IL34 exhibiting greater affinity and slower dissociation from CSF1R (23, 24), thus resulting in more sustained and stronger cell signaling (25) (reviewed in 26). CSF1 exclusively signals through the CSF1R, whereas IL34 also binds to the receptor-type protein tyrosine phosphatase-ζ (PTP-ζ) (27) and chondroitin sulfates, chiefly amongst these syndecan-1 (28). IL34-PTP-ζ interactions contribute to microglia homeostasis in the CNS whereas binding of IL34 to syndecan-1 serves as a means of regulating IL34 bioavailability and thus IL34-mediated CSF1R activation (27–29).

CSF1 and IL34 appear to confer at least partially non-overlapping roles in Mϕ development, homeostasis and polarization (29–31). For example, CSF1R signaling is essential for Langerhans cell (LC) development in most vertebrates (32) with IL34 being more critical to steady-state maintenance of LCs (30), while CSF1 contributes to LC development during skin inflammatory responses (33) (reviewed in 34, 35). The more recently discovered IL34 has also been linked with the development and maintenance of osteoclasts (36), microglia (37), and B-cell-stimulating mononuclear phagocytes (38). Indeed, human Mϕs differentiated with CSF1 and IL34 possess disparate polarization, with IL34-Mϕs exhibiting greater expression of IL10 and CCL17 (39) and greater resistance to HIV1 (40) and mycobacteria (41).

Mammalian CSF1-Mϕs have been implicated in tissue remodeling (42) and wound repair (43) and likewise, IL34-Mϕs promote healing (30) and collagen deposition (29). In turn, our previous studies indicate that frog (*Xenopus laevis*) CSF1- and IL34-Mϕs are morphologically and functionally distinct (44, 45). Both *csf1* and *il34* genes are expressed in healthy *X. laevis* skin (46), suggesting that CSF1- and IL34-Mϕs are both constitutively present in this tissue. Presently, we investigated the roles of these CSF1- and IL34-Mϕs in *X. laevis* scarless skin wound repair.

## 2 Materials and methods

### 2.1 Animal husbandry, wounding, and cytokine administration

*Xenopus laevis* juveniles (animals that have undergone metamorphosis within the past 30 days) were purchased from Xenopus 1 (Dexter, Michigan, USA) and reared in an aquatics laboratory in-house. Frogs were housed in an XR5 aquatic housing unit (IWAKI), and the water was filtered with carbon and sedimentation flow-through filtration systems. All animals were handled under the strict laboratory regulations of the Animal Research Facility at The George Washington University (GWU) and as per the GWU Institutional Animal Care and Use Committee regulations (Approval number A2023-044).

*X. laevis* juveniles (weighing approximately 2g) were anesthetized with 1g/L tricaine methanesulfonate (TMS), and sterile 2 mm biopsy punches (Royaltek) were used to excise both epidermal and dermal layers of dorsal skin while leaving the underlying musculature intact. Frogs were allowed to recover on moist filter floss and returned to water when recovered.

For Mϕ polarization experiments, frogs were subcutaneously injected at 3 days post-wounding (dpw) with 20μL (1μg/animal) of rCSF1, rIL34, or an equal volume of rctrl (generated as described below) in sterile amphibian-PBS.

### 2.2 Recombinant *X. laevis* CSF1 and IL34

We previously described the production of *X. laevis* recombinant (r)CSF1 and rIL34 (44, 45). Briefly, pMIB/V5His A (Invitrogen, Cat # V803001) insect cell expression constructs containing the signal peptide-cleaved representations of respective cytokine cDNAs were generated and transfected into Sf9 insect cells via Cellfectin II (Invitrogen, Cat # 10362100). Successfully transfected cells were selected using 100 μg/mL blasticidin, and expression was confirmed by Western Blot. Cultures were scaled to 300 mL, grown to confluency (5–6 x 10^6^ cells/mL), and the culture supernatants (sups) were dialyzed overnight at 4 °C against PBS, pH 8. Recombinant proteins were isolated from dialyzed sups using Ni-NTA Resin Beads (Thermo Scientific, Cat # PI88221), which were washed with 5 × 10 mLs of low-stringency wash buffer (50 mM Sodium Phosphate; 1 M Sodium Chloride; 40 mM Imidazole). Recombinant cytokines were eluted using 250 mM imidazole and were confirmed by Western blot against the V5 epitopes. Proteins were concentrated in polyethylene glycol at 4 °C to a volume of approximately 1mL and then dialyzed overnight at 4 °C against amphibian-PBS (APBS; 6.6g/L NaCl, 1.15g/L Na_2_HPO_4_, 0.4g/L KH_2_PO_4_), pH 7.4. Protein concentrations were determined by NanoDrop (RRID: SCR_023005). Proteins were stored at -20 °C in sterile aliquots until use.

A recombinant control (rctrl) was generated by transfecting Sf9 cells with an empty pMIB/V5 His A expression vector, selecting positive transfectants with blasticidin, scaling up these cultures, and processing the derived sups using the same approaches described above for rCSF1 and rIL34 production.

### 2.3 Histology and microscopy

Animals were euthanized by TMS overdose (5g/L) followed by cervical dislocation. The dorsal skins of individual animals were excised and fixed for 24 hrs in 10% NBF, transferred to 70% EtOH, and trimmed to a 6mm circle surrounding the wound (or a 6mm whole skin sample for controls). All tissues were processed at the Histopathology & Tissue Shared Resource at Georgetown University, District of Columbia. Tissues were embedded in paraffin, sectioned (5 μm), and stained with either hematoxylin and eosin, Picrosirius red (PSR; ScyTek, Cat #SRS250), or used for RNA *in situ* hybridization (RNA-ISH), as described below. Histology stains were performed according to the respective manufacturers’ instructions.

Hematoxylin and eosin and PSR-stained tissues were examined using either polarized light microscopy and/or brightfield microscopy using a Leica DMi8 Inverted Fluorescence Microscope at the GWU Nanofabrication and Imaging Center.

### 2.4 RNAScope fluorescent and chromogenic RNA-*in situ* hybridization

RNA-ISH was performed according to the instructions of the manufacturer, Advanced Cell Diagnostics (RRID: SCR_012481), documents USM322500, UM323100, and UM3400 and published by Wang et al. in 2012 (47). Briefly, tissues were fixed and sectioned as described above. The tissue was then deparaffinized, target retrieval was performed, and *X. laevis*-specific *il34* (Cat # 1220711-C1), *csf1* (Cat #1220701-C2)*, csf1r* (Cat #1310031-C3)*, mpo* (Cat # 1310041-C3), and *acta2* (Cat #1310051-C3) RNA probes were hybridized onto the tissue sections. *X. laevis*-specific positive control probes Cyclophilin B (*ppib;* Cat #580861-C2) and DNA-directed RNA polymerase II subunit RPB1 (*polr2a;* Cat #580841) and a negative control probe targeting *dapB* (Cat # 320871) from the soil bacterium *Bacillus subtilis*, were run in conjunction with each staining trial. Through a series of amplifications and developments, color-based signals were developed either using chromogenic stains or fluorescent probes. Images were acquired using a Leica DMi8 Inverted Fluorescence Microscope and analyzed using ImageJ, as described below.

### 2.5 Image analyses

All images were acquired with scale bars, which were used in ImageJ (RRID: SCR_003070) to assign scale to respective images and calculate areas of interest. Numbers of transcript-positive cells and quantities of transcripts were enumerated and quantified as proportions of tissue areas. When deemed helpful, RNA-ISH images were inverted using ImageJ software to optimally visually differentiate transcript staining from background staining.

Collagen I and III composition was quantified using ImageJ Trainable Weka Segmentation plugin, with the classifier trained to determine red, orange, yellow, green, and black (background) pixels within our PSR images. Some images were segmented into tiles for processing efficiency, and the same classifier was then applied to all respective images, and image averages were calculated. Color percentages as defined by the classifier were then quantified per target area and averaged across samples. Type I/type III collagen ratios were calculated by dividing the percent area of pixels with red, orange, or yellow (type I) by the percent area of pixels with green (type III) for each sample. To better visualize collagen organization, select polarized light images were also converted to black and white image casts using the built-in thresholding software.

### 2.6 RNA isolation and sequencing

For all experiments, tissues and cells were flash frozen in TRIzol™ reagent (Invitrogen, Cat #15596026) on dry ice and stored at -20 °C. RNA isolation was performed according to the manufacturer’s instructions, and samples were stored at -80 °C. Strand-specific RNA sequencing was performed by GENEWIZ (RRID: SCR_003177). Briefly, ribosomal RNA was depleted using a removal kit to enrich for messenger RNA and remove non-coding transcripts. Strand-specific RNA sequencing (RNA-seq) libraries were prepared using the NEBNext Ultra II Directional RNA Library Prep Kit (Cat # E7760S) or equivalent, following the manufacturer’s instructions, to preserve strand directionality. Library quality was confirmed via Qubit quantification and Bioanalyzer profiling. Libraries were sequenced on an Illumina NovaSeq 6000 Sequencing System (RRID: SCR_016387) platform using paired-end 150 bp reads, generating a minimum of 30 million reads per sample. Raw sequencing data was delivered in FASTQ format and subjected to downstream quality control and differential expression analysis using fastqc (v0.12.1) to view the quality of reads and fastp (v1.0.1) for adapter trimming with flag –detect_adapter_for_pe.

### 2.7 RNA sequencing analysis

Raw sequencing data was mapped to the RefSeq (RRID: SCR_003496) Xenopus_laevis_v10.1 GCF_017654675.1 assembly using Minimap2 (RRID: SCR_018550) with “-ax sr” and SAMTOOLS (RRID: SCR_002105) to produce a .bam file for each paired-end read set. An expression matrix was produced from all samples using featureCounts (RRID: SCR_012919) with parameters “–countReadPairs -B -t exon -g gene_id” using the GCF_017654675.1 genomic.gtf annotated from NCBI (RRID: SCR_006472). Downstream RNA sequencing analysis was performed using the iDEP: Integrated Differential Expression and Pathway analysis (RRID: SCR_027373) web application. Briefly, raw count data was pre-processed by removing lowly expressed genes and genes with 0.5 counts per million in at least 10 libraries were retained for further analysis. DESeq2 (RRID: SCR_015687) was then used to identify differentially expressed genes, where an adjusted p-value ≤ 0.05 was considered significant.

Data is available on NCBI under GEO accession number GSE306422 (https://www.ncbi.nlm.nih.gov/geo/query/acc.cgi?acc=GSE306422).

### 2.8 Statistical analyses

Where applicable, quantitative data was normalized to either the number of transcript-positive cells or the number of transcripts per unit area. Statistical analyses of all quantitative data (including Picrosirius red quantification data) were performed in RStudio (RRID: SCR_000432), using a one-way ANOVA followed by Tukey’s *post-hoc* test to determine pairwise significance. Data plotted in bar graphs is representative of two independent experiments. Error bars represent the standard error of the mean (SEM). RNA-seq expression data was averaged for each treatment across each time point, and the statistical significance of transcript expression across treatments was calculated in RStudio via pairwise Wilcox tests. The whiskers of box plots extend to the largest and smallest values within 1.5× the interquartile range from the box.

## 3 Results

### 3.1 Kinetics of juvenile *X. laevis* skin wound repair

**Figure 1:**
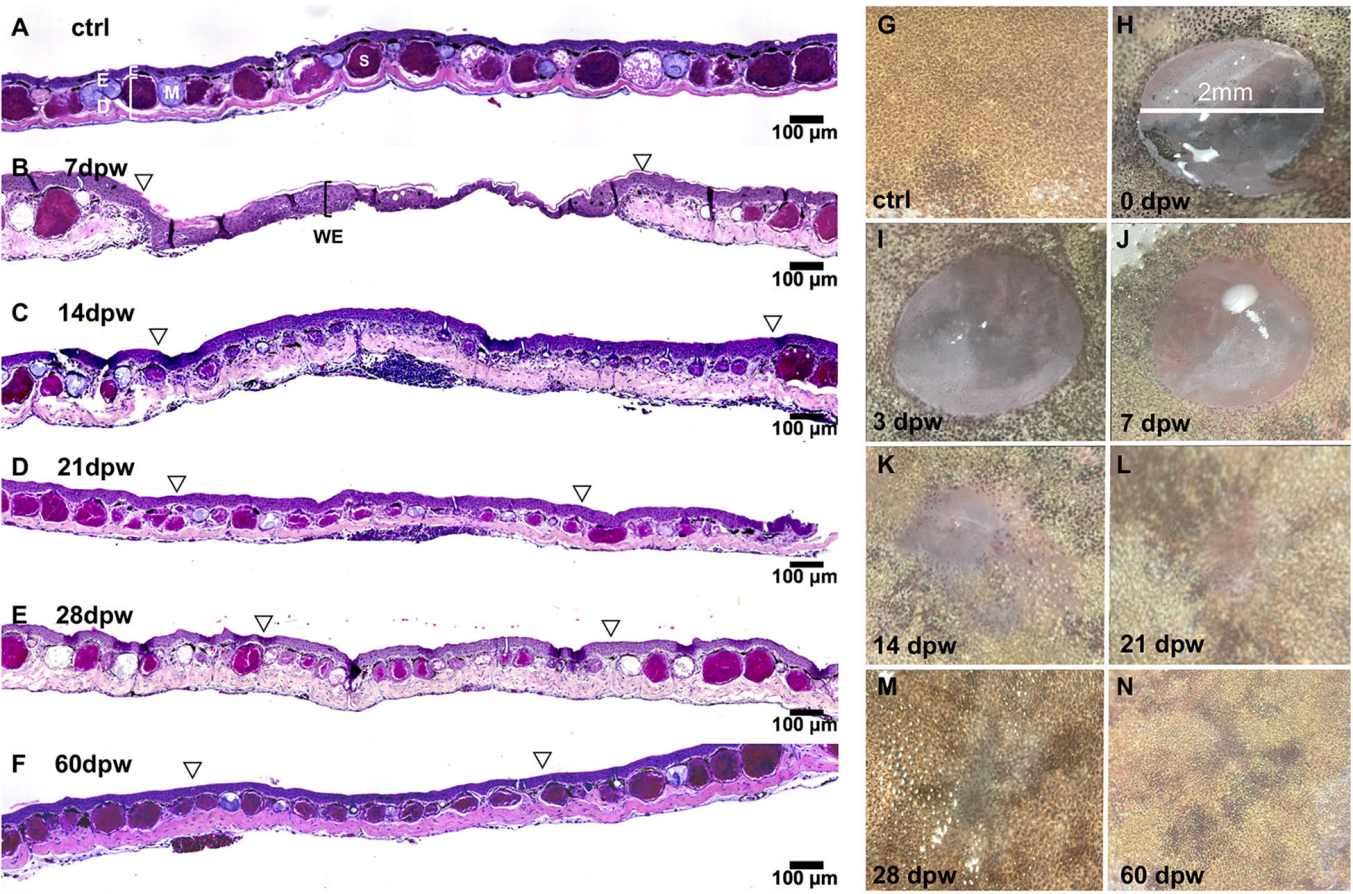
Juvenile *X. laevis* skin regeneration over time. **(A–F)** Hematoxylin and eosin (H&E) – stained skin sections from **(A)** unwounded controls (ctrl) and at **(B)** 7, **(C)** 14, **(D)** 21, **(E)** 28 and **(F)** 60 days post wounding (dpw). Open arrowheads mark wound edges. Control skin showing epidermis (E), dermis (D), and well-defined mucus (M) and serous (S) glands. WE, wound epidermis. **(G–N)** Top-down images of **(G)** control skin and wounds at **(H)** 0, **(I)** 3, **(J)** 7, **(K)** 14 **(L)** 21, **(M)**, 28 and **(N)** 60 dpw. Contrast and brightness adjusted uniformly across images.

We developed a biopsy punch approach to study *X. laevis* skin wound repair and examined the process over 60 days post wounding (dpw; localized epidermal and dermal dorsal skin damage; **Figure 1**). At 7 dpw we observed established wound epidermis (“WE”) several cells thick without distinguishable epidermal or dermal layers (**Figure 1B**). By 14 dpw demarcated epidermal and dermal layers were restored with skin glands beginning to form (**Figure 1C**) and by 21 dpw, the skin architecture was substantially restored, albeit with underdeveloped skin glands (**Figure 1D**). By 28 dpw, the repair process was substantially advanced and nearly complete by 60 dpw (**Figures 1E, F**). Top-down views of unwounded skin and healing wounds across the same time course complement our histological analysis (**Figures 1G–N**). At 60 dpw, the healed wound is indistinguishable from the surrounding tissue (**Figure 1N**), corroborating what we observe histologically.

### 3.2 Involvement of Mϕs, CSF1, and IL34 in regenerating frog skin wounds

**Figure 2:**
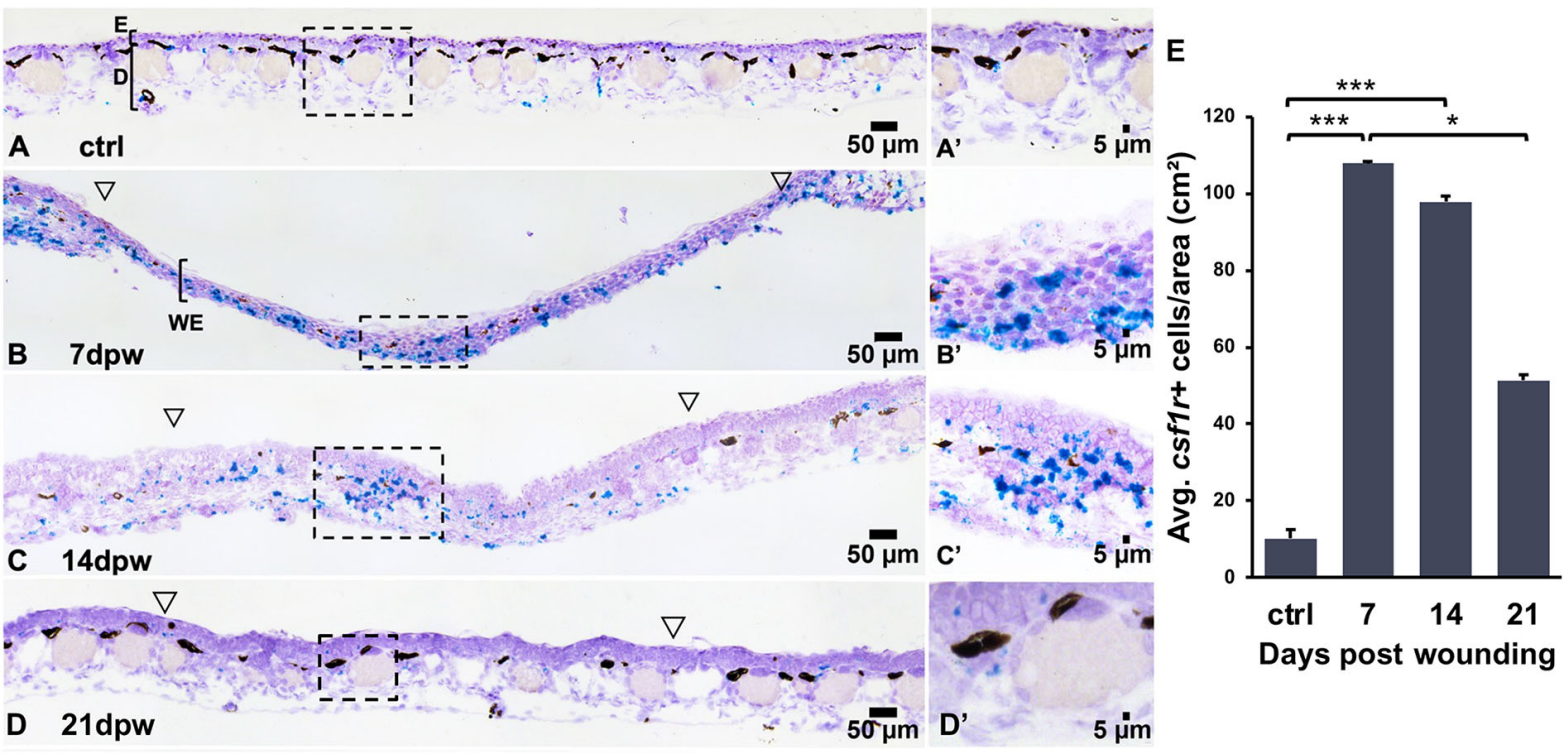
*Csf1r*^+^ macrophages accumulate transiently during wound repair. **(A–D′)** RNA *in situ* hybridization detecting *csf1r*^+^ cells (teal) in **(A, A′)** unwounded skin and at **(B, B′)** 7, **(C, C′)** 14 and **(D, D′)** 21 days post wounding (dpw). Open arrowheads mark wound edges, boxed regions indicate higher magnification in A′–D′. **(E)** Quantification of *csf1r*^+^ cell density (cells/cm²). Data are mean ± SEM (n = 4-5 biological replicates per group). One-way ANOVA with Tukey’s post hoc test; *p < 0.05, ***p < 0.001.

The colony stimulating factor 1 receptor (CSF1R) is indispensable to Mϕ biology, thus serving as a well-established marker of this leukocyte lineage (6). As such, we used *csf1r* expression to visualize Mϕs in regenerating *X. laevis* skin wounds. To characterize the kinetics of early *X. laevis* skin wound repair, full thickness wound areas from dorsal skins were collected at 0, 7, 14, and 21 dpw and Mϕs were visualized using RNA *in situ* hybridization (RNA-ISH), targeting *csf1r* transcripts (**Figure 2**). Unfortunately, the regenerating wound epithelia proved too fragile for histological processing of tissues earlier than 7 dpw. Our results indicate that Mϕs were present in healthy frog skins at relatively low levels, predominating in the dermal layers (**Figures 2A, A’**). Following skin wounding, high numbers of *csf1r+* cells could be seen infiltrating the WE at 7 dpw, with dense clusters appearing both within the wound bed and at the wound margins (**Figures 2B, B’**). With the WE thickening and dermal tissues beginning to reform by 14 dpw, the elevated *csf1r+* cell numbers persisted within regenerating wounds as clusters of Mϕs within the wound beds (**Figures 2C, C’**). By 21 dpw, wound *csf1r*+ Mϕ numbers had markedly declined, suggesting the resolution of Mϕ infiltration, as regeneration progressed (**Figure 2D, D’**). Quantification of these wound-infiltrating *csf1r+* cells confirmed these observations, wherein the numbers of *csf1r*+ cells per area were significantly elevated at 7 and 14 dpw compared to controls/unwounded skin (*p < 0.001*) and significantly decreased by 21dpw (*p < 0.05*, **Figure 2E**). Together, this data highlights a dynamic but transient Mϕ response in the regenerating skin, with maximal WE infiltration during the early and mid-phases of healing, followed by a decline as wound resolution occurs.

**Figure 3:**
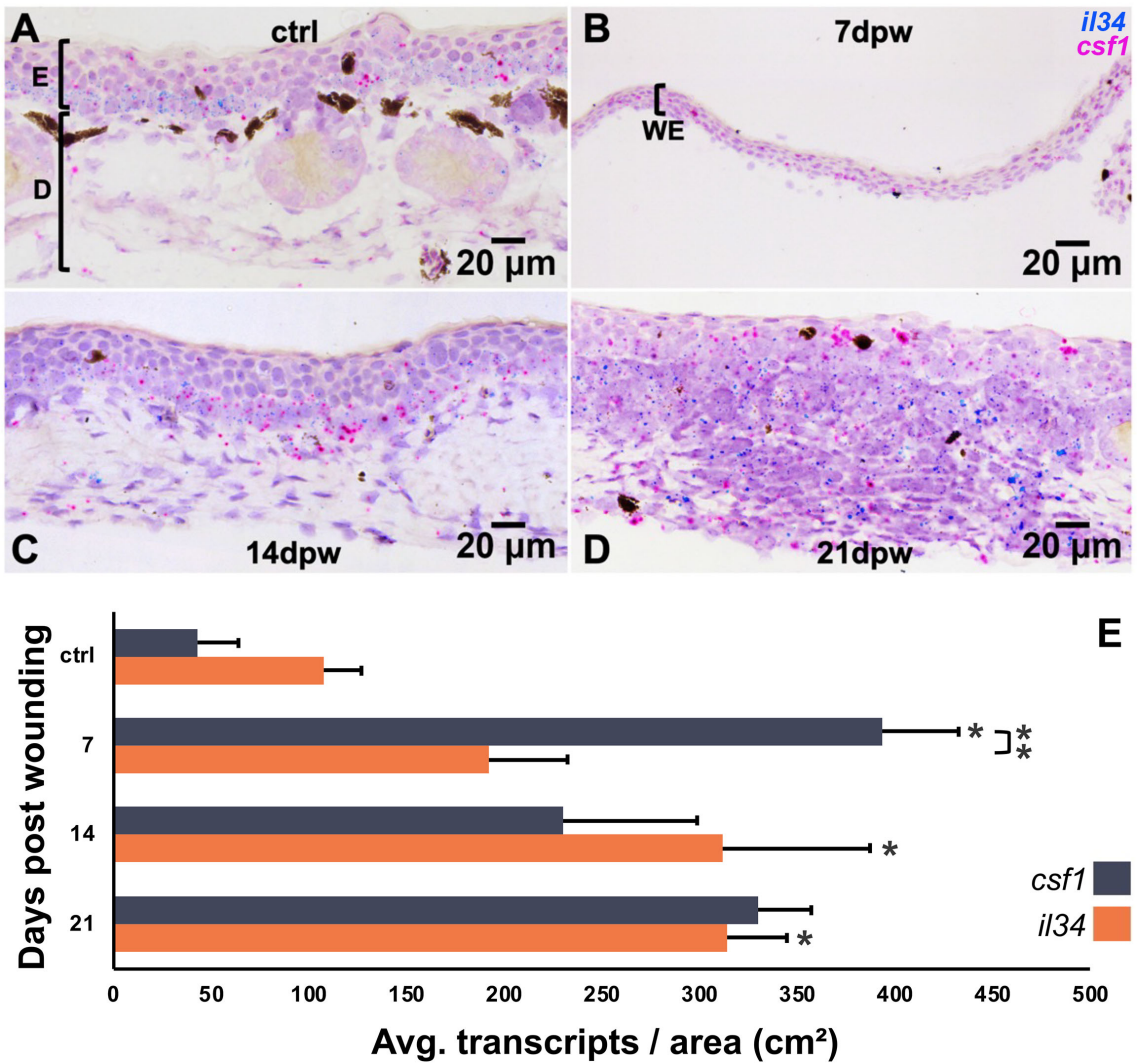
Expression of *csf1* and *il34* in regenerating skin wounds. **(A–D)** RNA *in situ* hybridization detecting *csf1* (magenta) and *il34* (blue/teal) transcripts in **(A)** unwounded skin, and skin at **(B)** 7, **(C)** 14 and **(D)** 21 days post wounding (dpw; counterstained with hematoxylin). WE, wound epidermis; E, epidermis; D, dermis. **(E)** Quantification of *csf1* and *il34* transcript density (transcripts/cm²). Data represent mean ± SEM (n = 4-7 biological replicates per group). One-way ANOVA with Tukey’s post hoc test; p < 0.05. Asterisks (*) indicate statistical significance from ctrl. Asterisks (**) above bar denotes significance between the groups indicated by the bar. * p < 0.05, ** p < 0.01.

To gain greater insight into Mϕ subtype involvement during skin regeneration, we next examined the expression patterns of principal Mϕ growth factors, colony-stimulating factor-1 (CSF1) and interleukin-34 (IL34) following skin wounding. Using RNA-ISH, we quantified transcripts per wounded area from tissue sections collected at 0, 7, 14, and 21 dpw (**Figure 3**). Transcript levels of *csf1* and *il34* in healthy, unwounded skin were low (**Figures 3A, E**), coinciding with our observations of relatively low proportions of Mϕs in healthy skin (**Figures 2A, E**). Expression of *csf1* was significantly elevated at 7 dpw compared to unwounded control levels (*p < 0.01*) and was significantly higher at that timepoint than *il34* expression (*p < 0.01*; **Figure 3E**). Expression of *csf1* declined thereafter, with significantly lower levels detected at 14 dpw from 7 dpw (*p < 0.05*, **Figure 3E**, significance not shown). In contrast, *il34* expression showed a more gradual increase following wounding, with the greatest expression observed 14 and 21 dpw (*p < 0.01* and *p < 0.05* compared to ctrl; **Figure 3E**). This suggests a shift in proportions of CSF1- to IL34-Mϕs with progressing wound repair.

### 3.3 Proportions of CSF1- and IL34-Mϕs influence the immune status of wounded skin

The transition from injury-induced inflammation to a resolution and repair phase is critical to prevent fibrotic scarring during wound healing (48). In mammals, pro-inflammatory Mϕs are present early on during wound formation/repair (5, 49, 50), producing inflammatory mediators (50). As inflammation resolves and wound healing transitions into proliferative and remodeling phases, Mϕs traditionally shift towards immune suppression and tissue remodeling phenotypes (5, 49). To define how CSF1- and IL34-Mϕs respectively influence the skin repair process, we wounded frogs as above and at 3 dpw subcutaneously injected them near the sites of injury with recombinant (r)IL34, rCSF1, or a recombinant control (rctrl). Dorsal skin wounds of juvenile frogs becomes re-epithelialized within 24–48 hours (51). This tissue is only several cells thick and is prone to tearing if manipulated. Thus, we waited 3 days post wounding before performing nearby subcutaneous injections. *Csf1r+* cell quantification via RNA-ISH indicated that cytokine treatment did not significantly increase the amount of Mϕs in the wounds (**Supplementary Figure 1**). We concluded that the following results reflect consequences of changing the polarization of Mϕs already present within skin wounds rather than recruitment of additional Mϕs.

**Figure 4:**
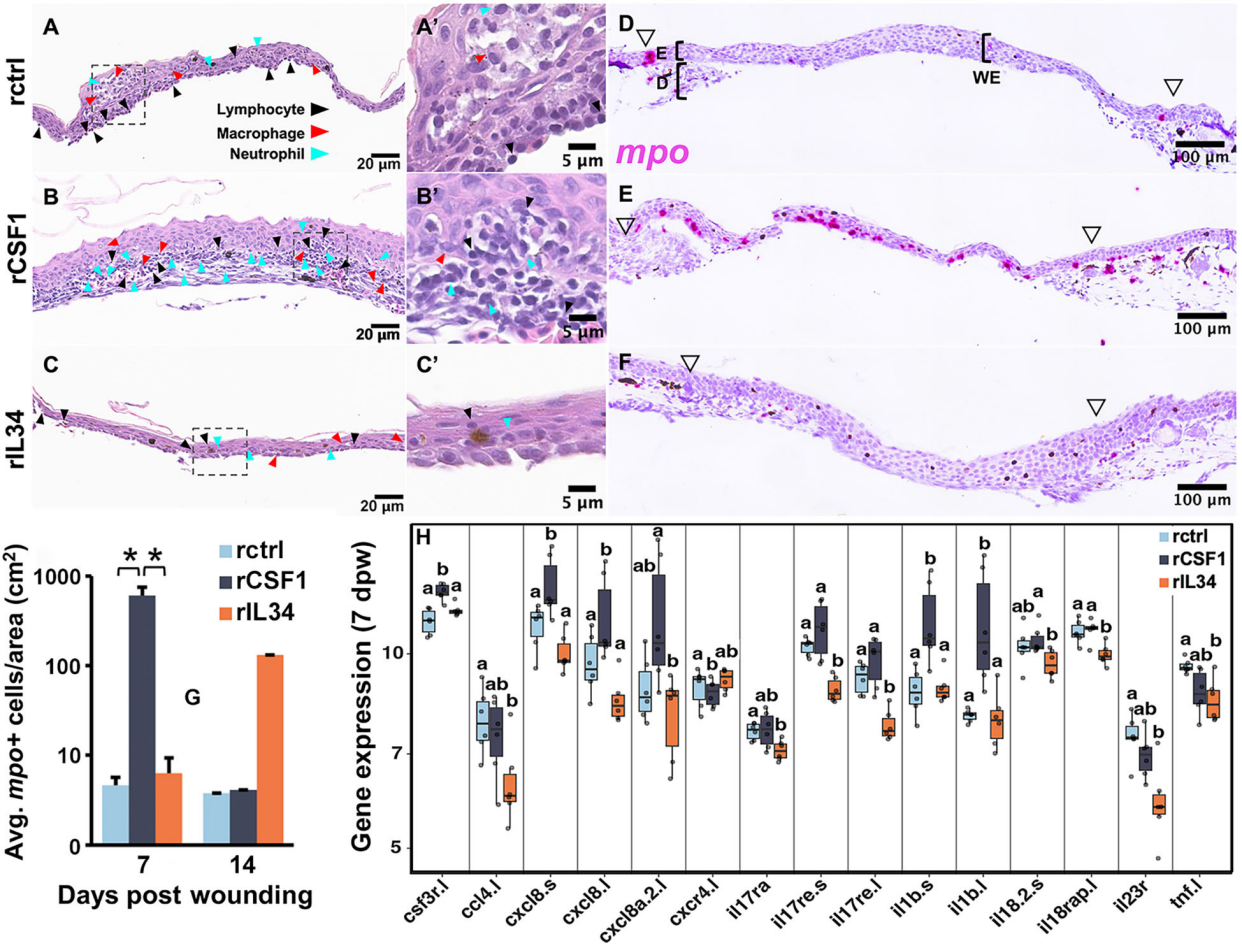
Analyses of histology and neutrophil infiltration of rCSF1- and rIL34-administered wounds. Histology, 7 days post wounding (dpw) of **(A, A′)** rctrl-, **(B, B′)** rCSF1-, and **(C, C′)** rIL34-administered wounds (cytokines administered 3 dpw). Inset panels **(A′–C′)** depict higher magnification. Lymphocytes (black arrows), macrophages (red arrows), and neutrophils (blue arrows) are indicated. **(D–F)** RNA *in situ* hybridization for mpo (magenta), visualizing neutrophil localization 7 days post wounding (dpw) in **(D)** rctrl-, **(E)** rCSF1- and **(F)** rIL34-administered wounds (cytokines administered 3 dpw). Open arrowheads mark wound edges. **(G)** Quantification of mpo⁺ cell density (cells/cm²) at 7 and 14 dpw. Data are mean ± SEM (n = 3-7 biological replicates per group). Statistical comparisons were performed using one-way ANOVA with Tukey’s post hoc test; *p < 0.05. **(H)** Expression analysis of inflammatory response genes at 7 dpw across treatments. Boxplots represent medians with interquartile range; letters indicate statistically distinct groups (*p* < 0.05, pairwise Wilcox test).

Because Mϕ recruitment to skin wounds peaked at 7 dpw (**Figure 2B**), we chose to focus our subsequent studies within and relatively soon after this timepoint. To this end, we next examined the wound histology of cytokine-administered animals, noting infiltration of leukocyte types therein at 7 and 14 dpw (**Figures 4A–C’**, **Supplementary Figures 2A–C**). At 7 dpw and compared to rctrl- and rIL34-treated wounds, the rCSF1-administered wounds possessed greater leukocyte infiltration, marked by greater proportions of neutrophils, lymphocytes, and Mϕs (**Figures 4B, B’**). At 14 dpw, we did not observe discernable differences in the histology of control and cytokine-treated wounds (**Supplementary Figures 2A–C**).

To corroborate the elevated neutrophil infiltration observed in rCSF1-administered wounds, we performed RNA-ISH analyses of myeloperoxidase (*mpo*), which is a defining neutrophil marker (14, 52, 53). Indeed, rCSF1-treated wounds had significantly higher proportions of *mpo+* cells at 7 dpw, compared to both control and rIL34-treated animals (**Figures 4D–G**). With respect to rctrl- and rCSF1-treatment groups, the rIL34-treated wounds exhibited greater numbers of *mpo+* cells at 14 dpw (**Figure 4G**, **Supplementary Figures 2D–F**), albeit not significantly so.

We next examined immune gene expression profiles of these cytokine-treated frog wounds during the repair process. The rCSF1-administered wounds possessed markedly greater pro-inflammatory gene expression at 7 dpw (**Figure 4H**). Amongst these was colony-stimulating factor 3 receptor (*csf3r.l*), a neutrophil growth factor receptor (54) and marker of frog neutrophils (55–57). Concurrently, the rCSF1-treated wounds exhibited greater expression of C-X-C motif chemokine ligand 8/interleukin-8 (*cxcl8*) genes, the products of which facilitate neutrophil recruitment (58, 59) as well as hallmark proinflammatory cytokines and receptors (*il1b.l/s*, *il17ra*, *il17re.l/s*; **Figure 4H**). By contrast, at 7 dpw, rIL34-treated wounds showed significantly decreased expression of the proinflammatory cytokines interleukin-1 beta (*il1β.l/s*) and tumor necrosis factor alpha (*tnf.l*). At 7 dpw, IL34-treated wounds also displayed reduced expression of receptors for interleukin-17, -18, and -23 (*il17ra, il17re.l, il18.2.s, il18rap.l, il23r*), neutrophil-associated genes *csf3r.l* and *cxcl8.l/s*, and the chemokine C-C motif chemokine ligand 4 (*ccl4*.*l*; **Figure 4H**). At 14 dpw, rCSF1-treated wounds continued to express greater levels of *csf3r* and *cxcl8.s* as well as the chemokine receptor, *cxcr4* when compared to controls (Supplementary **Figure 2G**). At this time, rIL34-administered wounds now also possessed greater mRNA levels of the *cxcr4* as well as several chemokine ligand genes (*ccl4.l/s, cxcl8.l/s, cxcl8a.2.l, cxcl13.l*; **Supplementary Figure 2G**), perhaps explaining the greater numbers of neutrophils seen in this treatment group at this time (**Figure 4G**, **Supplementary Figures 2D, F**). Taken together, these findings suggest that CSF1 and IL34 respectively promote and diminish wound inflammation, with rIL34 administration possibly having compensatory effects or changes to wound repair kinetics seen by 14 dpw.

### 3.4 Proportions of CSF1- and IL34-Mϕs in wounded skin influence myofibroblast activation, collagen deposition, and remodeling

**Figure 5:**
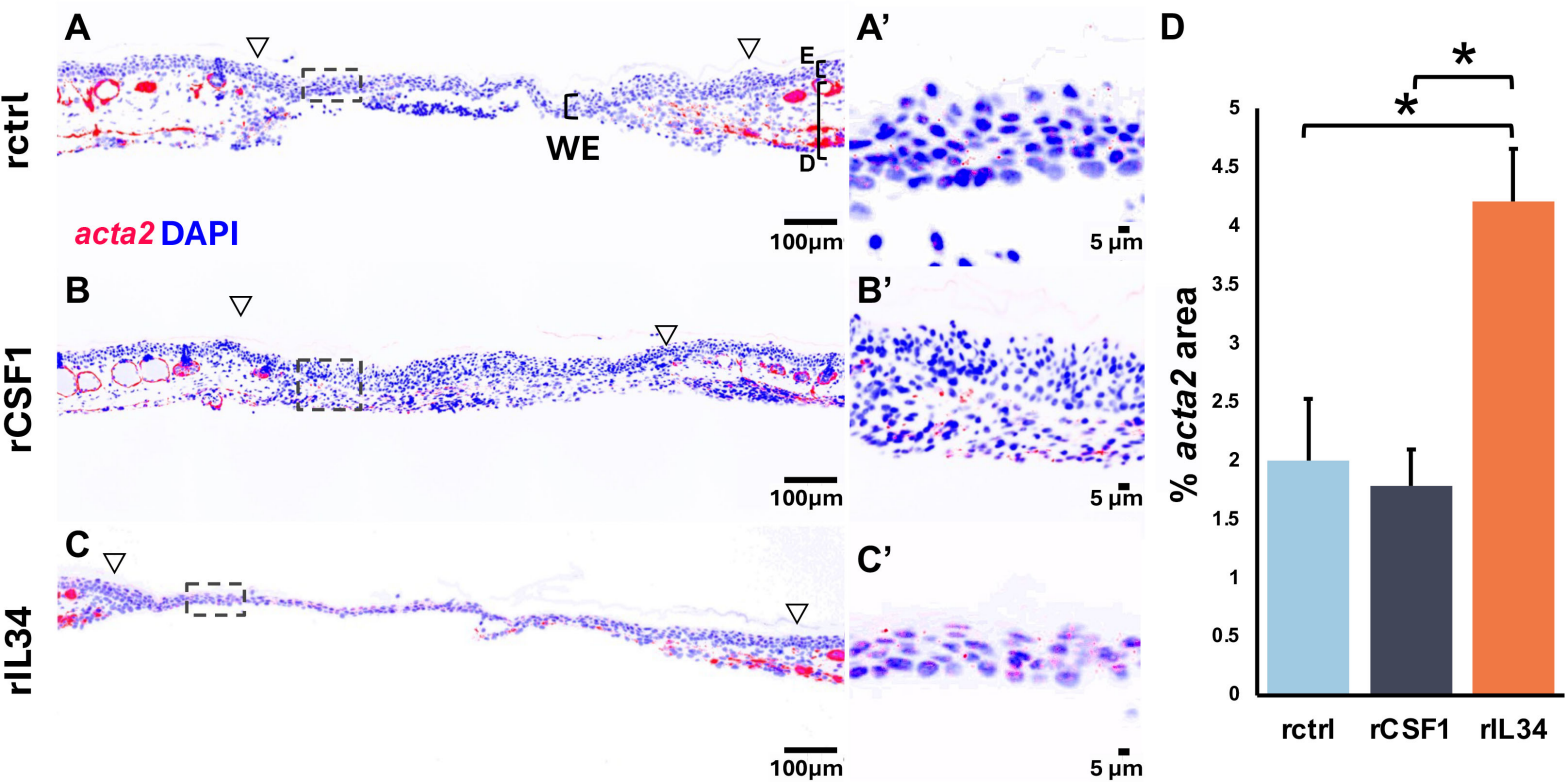
Myofibroblast accumulation in **r**CSF1- and rIL34-administered wounds. **(A–C′)** Immunofluorescence staining of (red) *acta2* and (blue) DAPI in **(A, A′)** rctrl, **(B, B′)** rCSF1, and **(C, C′)** rIL34 wounds at 7 dpw (cytokines administered 3 dpw). Open arrowheads mark wound edges, boxed regions indicate sections of higher magnification in **(A′–C′)**. **(D)** Quantification of *acta2*⁺ area (% total wound region, excluding gland-adjacent expression). Data represent mean ± SEM (*n* = 4-7 biological replicates per group). One-way ANOVA with Tukey’s *post hoc* test; *p < 0.05. Images were created using fluorescent microscopy and inverting uniformly in ImageJ.

To further understand the roles of CSF1- and IL34-Mϕs in wound repair, we characterized the kinetics of frog wound fibroblast activation. The activation of fibroblasts to the myofibroblast phenotype (*acta2+*) is a hallmark of wound healing across vertebrates (60), with a previous study (10) and the present work indicating that this is also true of *X. laevis* skin wounds. Compared to rctrl- and rCSF1-administered animals, frogs injected with rIL34 exhibited significantly more *acta2+* cells in their wounds at 7 and 14 dpw (**Figures 5A–D**, **Supplementary Figures 3A–D**), suggesting that IL34-Mϕs may be more important than CSF1-Mϕs in *X. laevis* skin wound myofibroblast activation. Notably, skin glands in and outside wounded skin also stained positive for *acta2* (**Figures 5A–C**, **Supplementary Figures 3A–C**), possibly owing to inherent roles for myofibroblasts in these structures.

**Figure 6:**
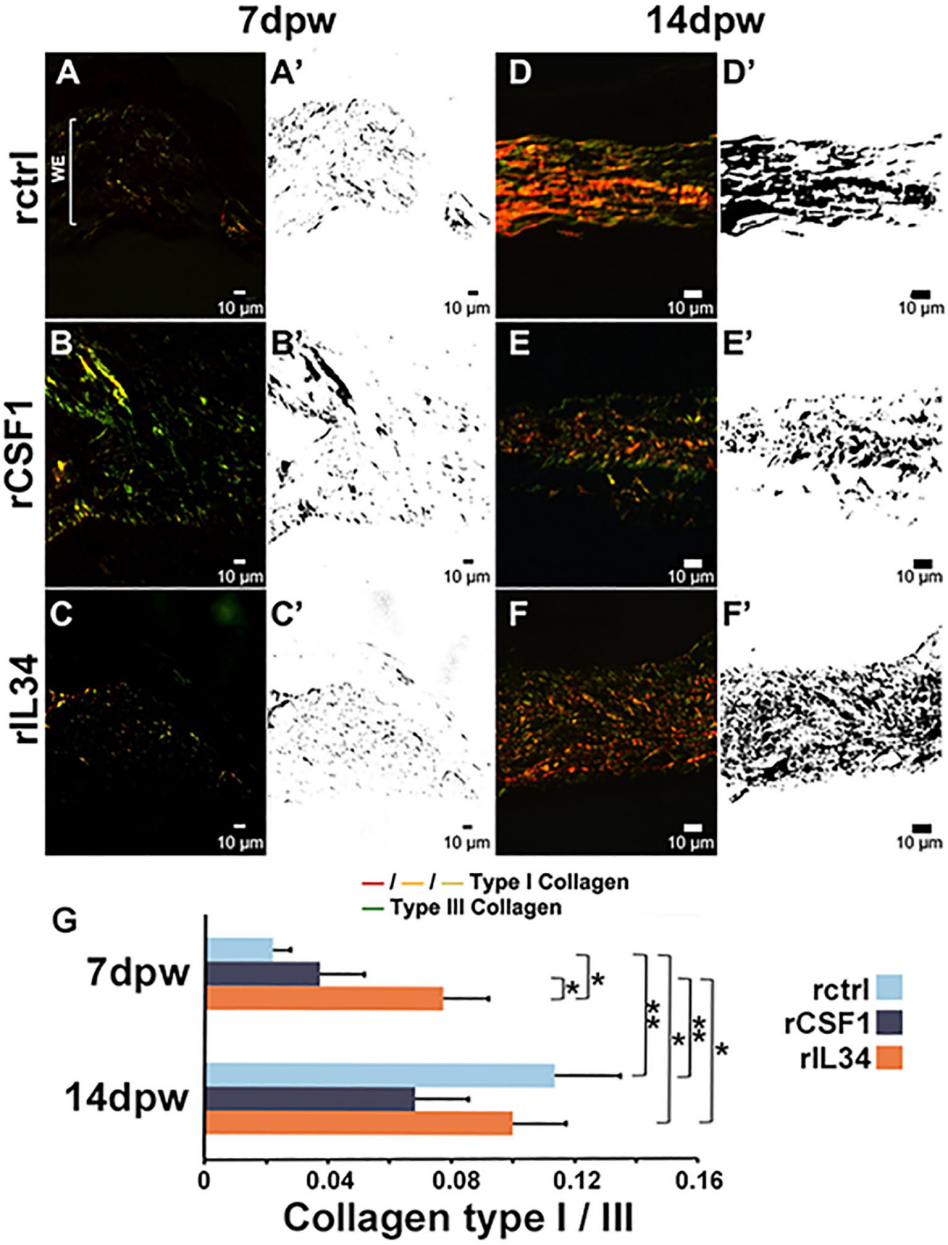
Collagen I and III deposition in rCSF1- and rIL34-administered wounds. **(A–C′)** Picrosirius red (PSR) staining of **(A, A′)** rctrl, **(B, B′)** rCSF1, and **(C, C′)** rIL34-administered wounds at 7 days post wound (dpw; cytokines administered 3 dpw), imaged with polarized light microscopy. Collagen I (red/orange/yellow) and collagen III (green) birefringence is shown; corresponding binary masks are in **A′–C′**. **(D–F′)** PSR staining of **(D, D′)** rctrl, **(E, E′)** rCSF1, and **(F, F′)** rIL34 wounds at 14 dpw with corresponding masks **(D′–F′)**. **(G)** Quantification of collagen I/III ratios at 7 and 14 dpw. Data represent mean ± SEM (*n* = 7-12 biological replicates per group). One-way ANOVA with Tukey’s *post hoc* test; *p < 0.05, **p < 0.01.

Myofibroblast-mediated wound repair is marked by collagen deposition and remodeling (4, 9, 61) while mammalian skin is predominated by collagen types I and III (43, 62, 63). In turn, analysis of the collagen I/III ratio is frequently used to assess scarring and fibrosis in mammalian wound healing studies (64). Because myofibroblast activation drives this process, we next examined collagen deposition in rCSF1- and rIL34-skewed skin wounds during repair (**Figures 6A–F’**). When we examined the ratios of collagen I to collagen III deposition in the healing skin, we found that at 7 dpw the rIL34-administered animals possessed significantly higher collagen I/III ratios compared to control and rCSF1-treated animals (*p < 0.05*, **Figures 6A–C, G**), suggesting that IL34-Mϕs promote type I collagen deposition, presumably at least in part via myofibroblast activation. While the collagen I/III ratios were less prominent in rCSF1-administered wounds than in rIL34-treated wounds at 7 dpw (**Figure 6G**), those tissues displayed greater overall amounts of both collagen types, but especially type III (**Figure 6B**).

By 14 dpw, all treatment groups had greater collagen deposition, with control and rIL34-treated wounds exhibiting greater (not significantly so) collagen I/III ratios than rCSF1-administered wounds (**Figures 6D–G**). Moreover, at 14 dpw the patterns of the remodeled collagen were markedly different across these treatment groups (**Figures 6D–F’**). The control wounds displayed mostly parallel collagen fibers across the wound beds by 14 dpw, consistent with what is seen at this stage in other vertebrates with superior regenerative capacities (65) (**Figures 6D, D’**). The rCSF1-treated wounds exhibited dense, fragmented collagen with irregular orientation, indicative of disorganized deposition/remodeling and reminiscent of what is typical of pro-inflammatory milieu (65, 66) (**Figures 6E, E’**). In contrast, rIL34-treated wounds showed dense collagen deposition with fibers that were more interwoven and heterogeneous in orientation, suggesting accelerated but remodeling-competent matrix assembly (**Figures 6F, F’**).

**Figure 7:**
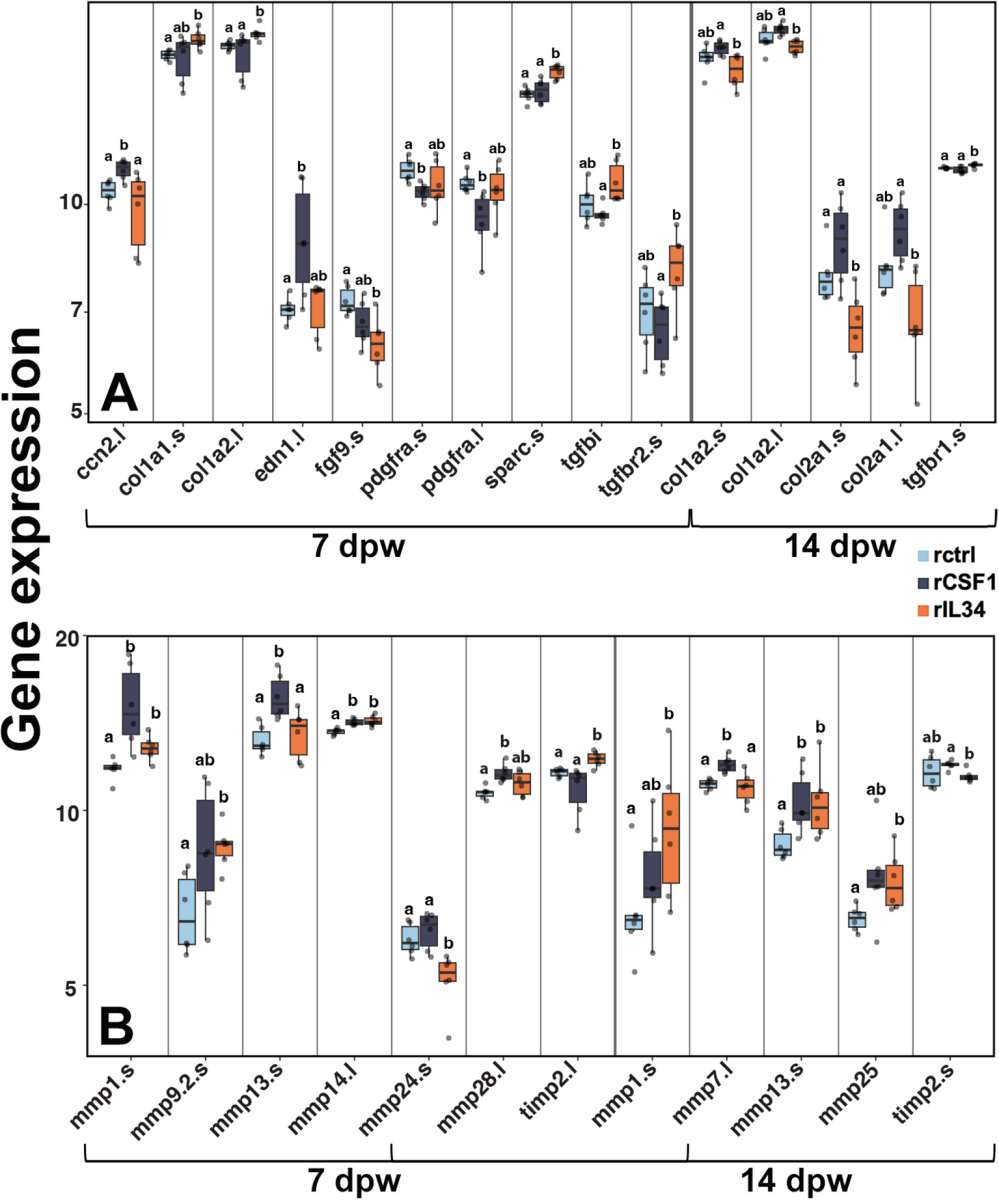
Gene expression in rCSF1- and rIL34-administered wounds. **(A)** RNA-seq expression analysis of ECM- and fibrosis-associated genes at 7 and 14 dpw across treatments. **(B)** RNA-seq expression analysis of matrix metalloproteinases (MMPs) and their regulators (TIMPs) at 7 and 14 dpw across treatments. Boxplots show median, interquartile range, and statistical groupings, letters denote significance; *p* < 0.05.

Our transcriptomic analysis supported the above observations. For example, rIL34-treated wounds exhibited significantly greater expression of collagen type I alpha 2 chain (*col1a2.l/s*) at 7 dpw (**Figure 7A**), consistent with their greater I/III ratios (**Figure 6G**). In line with greater myofibroblast activation (**Figure 5**), rIL34-treated wounds also had greater expression of secreted protein acidic and cysteine rich (*sparc.s*; **Figure 7A**), the product of which contributes to myofibroblast differentiation and regulation of collagen-remodeling (67). The gene encoding transforming growth factor beta-induced protein (*tgfbi*), a downstream effector of TGFβ1, involved in collagen fiber organization and myofibroblast contractility (68) was likewise upregulated in rIL34-treated wounds, as was *tgfbr2.s* (**Figure 7A**), the ligand for which is prominently involved in wound repair and fibrosis (69). Moreover, fibroblast growth factor-9 (FGF9) is known to inhibit TGFβ1-induced myofibroblast differentiation (70), and thus our observation that *fgf9.s* expression was decreased in the rIL34-treatment group, also supports greater fibroblast activation at 7 dpw in this group (**Figure 7A**). Reduced platelet derived growth factor (PDGF) signaling is a hallmark of scarless healing (71) and so the decreased expression of several PDGF-related genes (*pdgfa.l, pdgfc.s*, and *pdgfrb.s*) in rIL34-treated wounds likewise corroborates the possible role of IL34 in regenerative repair (**Figure 7A**). Elevated connective tissue growth factor (*ccn2*) and endothelin-1 (*edn1.l*) levels have been respectively linked to fibrosis (72) and vascular dysfunction (73), so it is notable that these transcripts were elevated in rCSF1-treated wounds but diminished in rIL34-administered wounds (**Figure 7A**). By 14 dpw, the rIL34-treated wound had significantly diminished expression of collagen genes, *col1a2.s/l* whereas the rCSF1-treated wounds possessed marginally increased *col1a2.s/l* expression (**Figure 7A**).

Extracellular matrix remodeling by matrix metalloproteinases (MMPs) and their regulation by tissue inhibitor of metalloproteinases (TIMPs) is a key feature of wound repair (48, 74) and this process is integral to collagen remodeling and amphibian regeneration (75–77). Our results showed that at 7 dpw, rCSF1-treated wounds possessed elevated expression of genes encoding pro-fibrotic MMPs (*mmp13.s*, *mmp28.l*) *(*75, 76, 78) as well as the pro-collagen I remodeling *mmp14.l* (66, 79) (**Figure 7B**). The rIL34-treated wounds showed significantly increased expression of *mmp9* and *mmp14*, the former known to be important in regeneration (79, 80) (**Figure 7B**). The rIL34-treated wounds also had significantly reduced expression of the pro-fibrotic *mmp24* (81) and upregulated expression of *timp2.l*, a prominent MMP inhibitor (69) (**Figure 7B**). Both treatment groups exhibited elevated levels of *mmp1.s* when compared to controls, albeit more modestly so in rIL34-treated wounds (**Figure 7B**). At 14 dpw, CSF1-enriched wounds maintained high expression of *mmp7* and *mmp13*, which are known to be upregulated during inflammation (72). Conversely, rIL34-enriched wounds expressed greater levels of *mmp1* and *mmp25*, the latter being associated with angiogenesis (79, 82) (**Figure 7B**). These findings demonstrate that rCSF1 and rIL34-polarization of regenerating wounds culminated in distinct MMP signatures despite comparable collagen I/III ratios at 14 dpw.

## 4 Discussion

By two months post injury, juvenile *X. laevis* skin wounds are entirely regenerated to a point of being indistinguishable from the surrounding tissue, both histologically and macroscopically (83). At this time the afflicted skin possesses fully reformed epidermal and dermal layers, along with regenerated glands, which are albeit smaller and fewer in number than those in the surrounding unwounded skin (83). In contrast, adult *X. laevis* exhibit fibrotic repair of their skin wounds, characterized by irregularly organized collagen fibers (10). Much remains to be discerned about the immune determinants of juvenile versus adult *X. laevis* wound repair capacities. The results presented here indicate that *X. laevis* Mϕ subsets are intimately involved in the juvenile *X. laevis* skin wound repair.

Mϕs are rapidly recruited to *X. laevis* juvenile skin wounds and remain within the regenerating tissues for up to three weeks. This is reminiscent of the mammalian phases of wound healing, wherein Mϕs are essential for early inflammation, debris clearance, and signaling to downstream effectors like fibroblasts (12). In turn, our expression analysis of *csf1* and *il34*, as well as our Mϕ polarization studies indicate that CSF1- and IL34-Mϕs play disparate and temporally regulated roles in wound repair. Altering the relative proportions of CSF1- and IL34-Mϕs in the frog wounds resulted in profound consequences to the immune status of those wounds. Whereas rIL34-treatment manifested in reduced gene expression of key pro-inflammatory mediators, rCSF1 stimulation increased the expression of several hallmark inflammatory genes alongside increased neutrophil infiltration into the wound beds. Accordingly, we postulate that CSF1-Mϕs predominate the initial inflammatory responses, whereas IL34-Mϕs likely promote wound repair through fibroblast activation, altered collagen deposition, and advanced remodeling later in the repair process. It is of note that in mammals, IL34 facilitates steady-state maintenance of epidermal skin Langerhans cells (LCs) (30) whereas CSF1 contributes to LC production during skin inflammatory responses (33). In turn, our past studies indicate that the *X. laevis* CSF1-Mϕs may be more inflammatory than IL34-Mϕs in certain contexts (45, 84), while there is a growing body of literature indicating that in mammals, IL34 is produced in tolerogenic contexts by T regulatory (Treg) cells (85). Future studies employing repeated injections or sustained formulated release of rCSF1 and rIL34 will grant added perspectives on these respective Mϕ subsets and on the utility of such reagents to therapeutic applications.

In mammals, cutaneous injury triggers the rapid recruitment of circulating monocytes into wound beds, which extravasate from blood vessels, enter the wound area, and differentiate into inflammatory Mϕs or monocyte-derived dendritic cells (MoDCs) (86). These recruited cells are reinforced by locally expanding skin-resident dermal Mϕs and LCs (58, 87), which also contribute to wound surveillance and orchestration of repair processes. Resident tissue Mϕ and DC precursors are seeded into tissues like skin during embryonic development and self-renew therein, independently of blood-derived monocytes (88). This includes mammalian LC precursors, which repopulate LCs at steady-state (88). Though it is not clear from our analysis where the Mϕs involved in amphibian wound healing originate, we speculate that these effector Mϕs comprise of both tissue-resident and recruited, hematopoietic progenitors-derived populations. recruited. Considering the number of Mϕs decreases with frog wound repair, concurrent with changes in *il34* and *csf1* gene expression, we anticipate that some of these cells leave the wounds or undergo apoptosis. However, other Mϕs likely remain, shifting from CSF1-polarized to IL34-polarized subtypes, possibly resembling the shift from pro-inflammatory to anti-inflammatory Mϕ phenotypes seen in healing mammalian wounds (89). It will be interesting to see the extent to which this CSF1/IL34 wound repair dichotomy is evolutionarily conserved.

Fibroblasts are critical to wound repair (60, 90, 91), differentiating into myofibroblasts upon activation and upregulating their expression of alpha-smooth muscle actin (*acta2/acta2*) (4, 17, 90). Fibroblasts are thought to migrate into wounds from surrounding tissues (92), though it is not clear in either frogs or mammals, what proportion of these cells come from neighboring healthy skin versus other sources. Myofibroblasts synthesize and deposit collagen types I and III (90), causing wound contraction and closure (5, 64) as well as scar formation and fibrosis in mammalian wound healing (4, 34). Mammalian scar tissue formation reflects higher collagen I/III ratios (i.e. higher levels of collagen I), with normal and hypertrophic scars possessing a 6:1 ratio and keloid scars showing a staggering 17:1 ratio (64). During mammalian wound healing, thin collagen III fibers are deposited first, followed by the thicker collagen I fibers, as scars form (90). In contrast, regenerating amphibians actively suppress type I collagen deposition early on during wound repair, while type III collagen is deposited in low amounts as loose fibers (93). As amphibian regeneration progresses, collagen I is deposited considerably more slowly than in mammals, preventing fibrosis and scar formation. We anticipate that the greater type I collagen deposition and increases in *acta2+* myofibroblasts seen in rIL34-treated wounds reflect the roles of IL34-Mϕs in later, reparative phase of the repair process. Consistent with this notion, while rIL34-administration increased *col1a* gene expression, it also resulted in reduced inflammatory gene expression, suggesting a transition towards repair. Conversely, rCSF1-treatment elevated pro-inflammatory transcripts and total collagen deposition. This interpretation is further supported by the divergent expression of matrix metalloproteinases (MMPs) and a regulator, tissue inhibitor of metalloproteinases-2 (TIMP2), as these are the principal mediators of collagen turnover. Cytokine treatments produced distinct MMP profiles that paralleled the elicited collagen deposition phenotypes. At 7 dpw, rCSF1-treated wounds possessed elevated MMP13 and MMP28, which are respectively associated with granulation and fibrosis (81, 94). Both cytokine treatment groups upregulated the pro-fibrotic MMP1 (76), but only rIL34-treated wounds had increased TIMP2 expression. We anticipate that the high MMP1, MMP13 and MMP28 expression in the rCSF1-treated wounds is more likely to result in fibrosis and scar formation. Conversely, modestly increased MMP1 together with high expression of the pro-regenerative MMP14, MMP25 and TIMP2 concurrent with reduced MMP24, seen in rIL34-treated wounds, likely results in more controlled collagen remodeling and is less likely to lead to scar formation. At 14 dpw, in addition to MMP13, rCSF1-treated wounds also exhibited elevated expression of MMP7, which has been associated with delayed healing (87). The rIL34-administered wounds possessed elevated MMP1, but also MMP25 expression, reminiscent of MMP profiles associated with localized collagen trimming (79, 95, 96). It will be interesting to learn the precise kinetics with which disparate Mϕ subsets control deposition and remodeling of extracellular matrices.

TGFβ signaling is a hallmark of *X. laevis* regeneration (97), and rIL34-treated wounds possessed greater expression of multiple TGFβ downstream effectors including *col1a2*, *sparc*, *tgfbi*, and *tgfbr2l*. The increase in *tgfbr2l.s* is especially compelling as TGFβR2 is the prominent receptor in TGFβ signaling (97). SPARC and TGFβI, both upregulated in rIL34-treated wounds, are key regulators of collagen organization and fibroblast contractility (98). Their increased expression, alongside more robust myofibroblast recruitment/activation, likely contributes to the higher collagen I deposition seen in rIL34-treated wounds. These observations support our notion that IL34-Mϕs promote wound healing following the inflammatory phase of the repair response. Pertinently, studies looking at tail and limb regeneration in *X. laevis* have indicated that early-phase treatment of wounds with anti-inflammatory agents enhance regenerative capacities (99).

It is speculated that mucosal tissue possesses traits that enable regeneration, which is why oral mucosa in mammals can heal with little to no scar formation (87). Mammalian oral mucosa wound repair leads to increased release of chemokine CCL5, a robust early influx of neutrophils and Mϕs, and higher levels of IL6, IL1β, and TNFα (87). This presents an interesting comparison, as these overlap with the expression profiles seen in the wounds of both the control and rCSF1-treated animals. Even though frog skin serves as both a skin and a mucosal barrier, our previous work suggests that other frog skin-resident immune cells, at least in some contexts, more closely resemble mammalian skin-resident over their mucosal counterparts (100). The thus far characterized frog inflammatory response elicited during wounding also closely resemble those seen in human wounds (101).

While our polarization studies indicate potentially disparate roles for CSF1- and IL34-Mϕs during regenerative skin repair, much remains to be learned about how these respective subsets confer changes to aspects of wound repair such as recruitment and activation of distinct fibroblast subsets as well as deposition and remodeling of extracellular matrix components. We did not see increased Mϕ infiltration of wounds treated with rCSF1 or rIL34, suggesting that the observed effects are due to polarization rather than expansion of the respective Mϕ subset. Unfortunately, our attempts to deplete these Mϕ types within wounds by administering purified polyclonal antibodies against frog rCSF1 and rIL34 were not successful. While we believe our present findings to be compelling, we also feel that further resolution of when and how CSF1- and IL34-Mϕs contribute to scarless wound repair needs to be achieved through future studies using Mϕ-subset-specific depletion or knock-out approaches.

The present findings represent a window into the roles of frog Mϕ subsets in scarless wound repair. However, much remains to be discerned regarding the coordinated and kinetic involvement of these and other myeloid populations in frog skin regeneration and how these cells and processes relate to those taking place in mammals. We believe that studies akin to those reported here will bridge that gap in our understanding of the determinants of successful wound repair.

## Data Availability

The datasets presented in this study can be found in online repositories. The names of the repository/repositories and accession number(s) can be found below: https://www.ncbi.nlm.nih.gov/geo/query/acc.cgi?acc=GSE306422, GSE306422.
