# Supplementary material for: The contribution of amphibian macrophage subsets to scarless regeneration of skin wounds
Source: Front Immunol. 2025 Dec 11;16:1713361. doi: 10.3389/fimmu.2025.1713361 (PMC12738165; doi:10.3389/fimmu.2025.1713361)
Supplement: Supplementary file 1 [file DataSheet1.docx]

Supplementary Material

# Supplementary Figures

# **
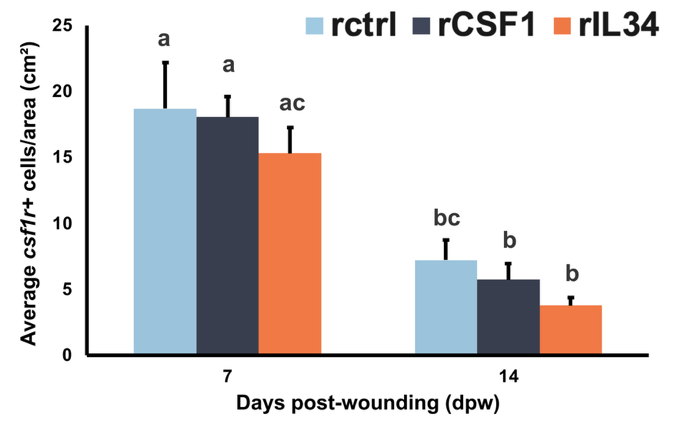
**

**Supplementary Figure 1. Quantification of** csf1r**⁺ cells in** rCSF1- and rIL34-administered wounds**.** Average density of csf1r⁺ cells (cells/cm²) at 7 and 14 days post wounding (dpw) in rctrl-, rCSF1-, and rIL34-administered wounds (cytokines administered 3 dpw). Data represent mean ± SEM (n = 6-8 biological replicates per group). One-way ANOVA with Tukey’s post hoc test, letters denote significance groupings; p < 0.05.

**
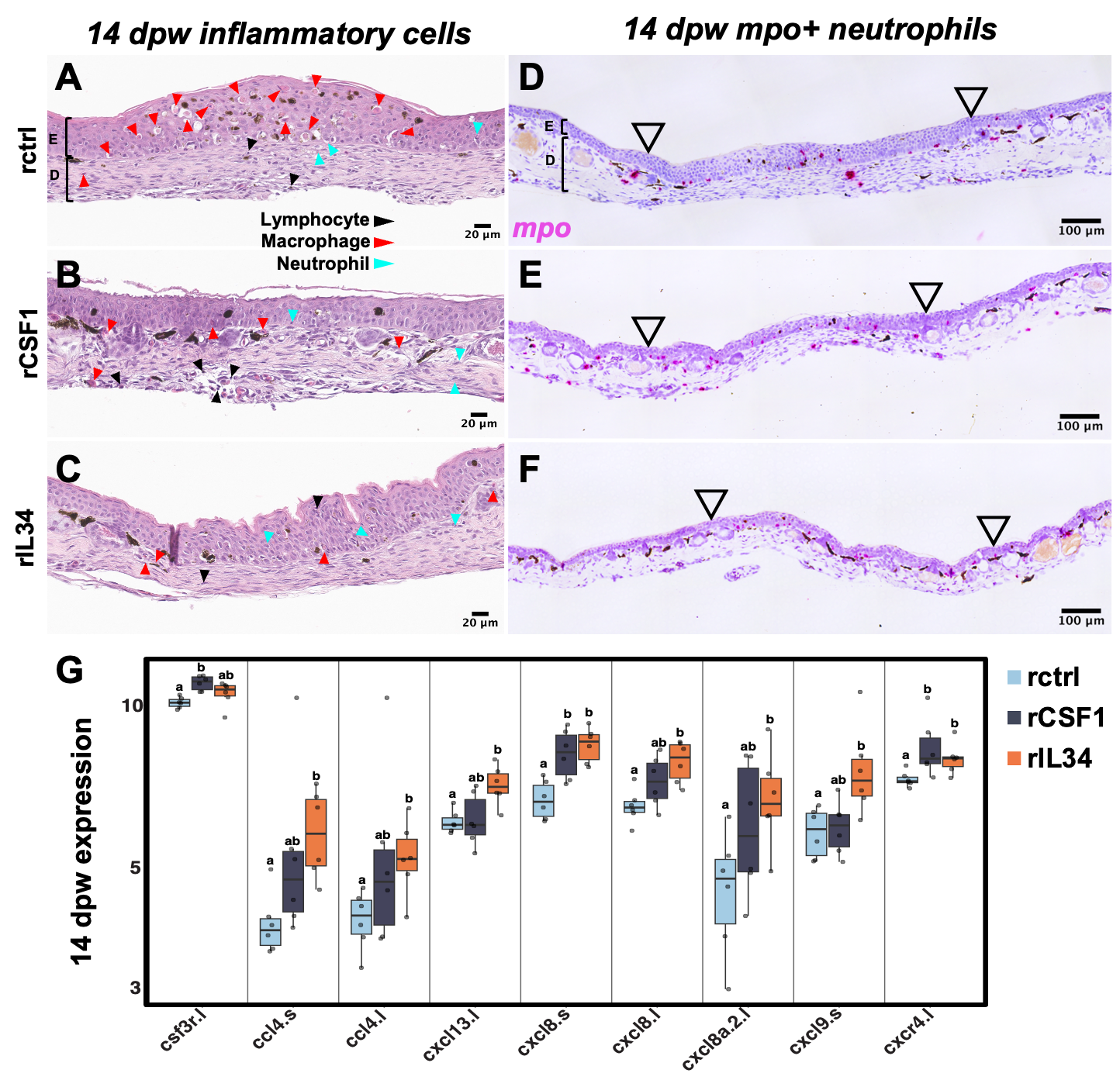
**

**Supplementary Figure 2. Inflammatory cells and neutrophils in** rCSF1- and rIL34-administered wounds **at 14** days post-wound (dpw)**.** (A–C) Histological sections of (A) rctrl-, (B) rCSF1-, and (C) rIL34-administered wounds 14 dpw (cytokines administered 3 dpw). Lymphocytes (green arrows), macrophages (red arrows), and neutrophils (blue arrows) are indicated. (D–F) RNA *in situ* hybridization detecting mpo transcripts (magenta) in (D) rctrl- (E) rCSF1- and (F) rIL34-administered wounds. Open arrowheads mark wound edges. (G) RNA-seq expression analysis of inflammatory response genes at 14 dpw. Boxplots show median, interquartile range, and statistical groupings, letters denote significance; p < 0.05. Contrast and brightness adjusted uniformly across images.


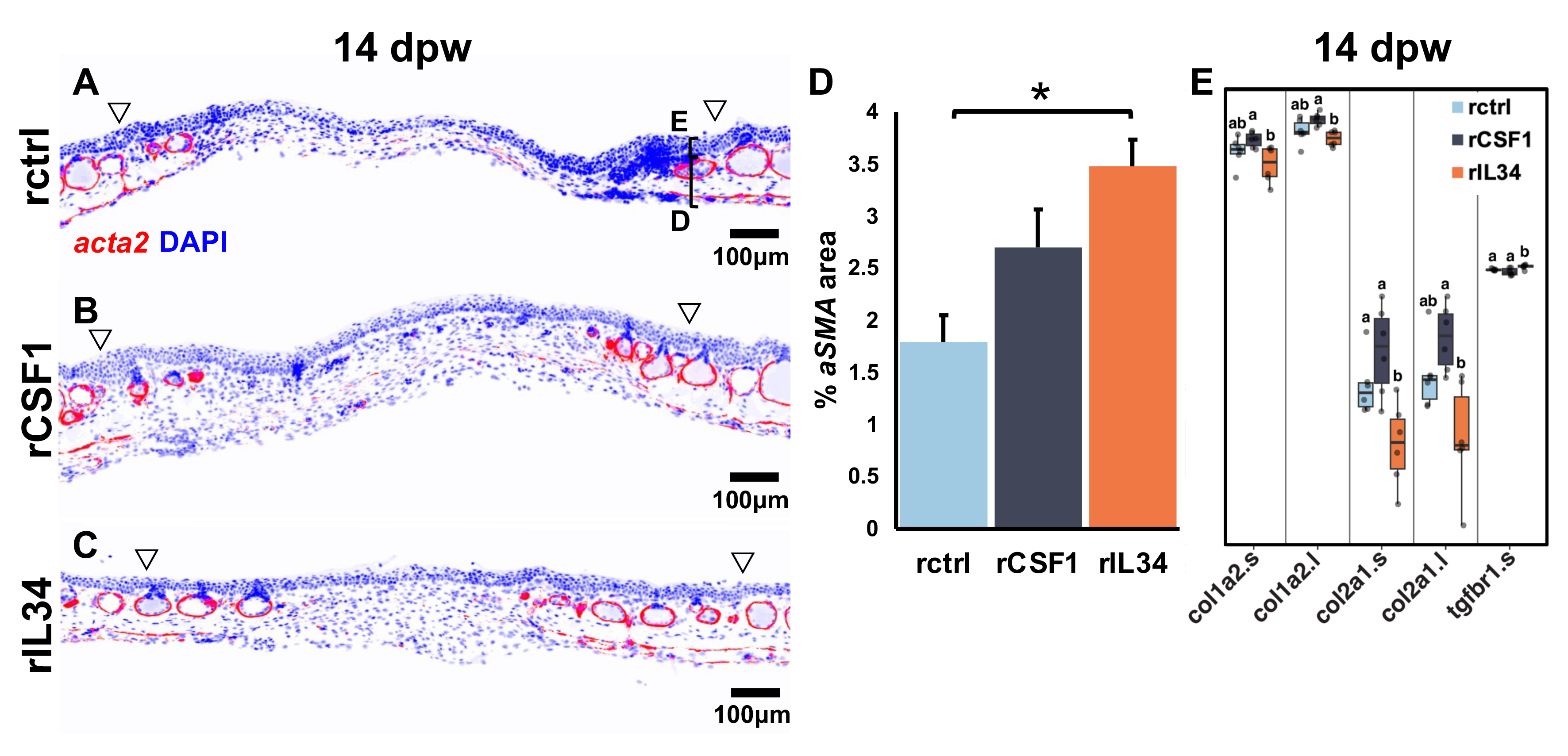


**Supplementary Figure 3. Myofibroblast activation and fibrosis-associated transcripts in** rCSF1- and rIL34-administered wounds at **14 dpw.** (A–C) Immunofluorescence staining of *acta2* (red) and DAPI (blue) in (A) rctrl-, (B) rCSF1- and (C) rIL34-administered wounds at 14 dpw (cytokines administered 3 dpw). Open arrowheads mark wound edges. (D) Quantification of *acta2*⁺ area (% total wound region, excluding gland-adjacent expression). Data represent mean ± SEM (n = 6-7 biological replicates per group). One-way ANOVA with Tukey’s post hoc test; p < 0.05. (E) RNA-seq expression analysis of fibrosis-associated genes at 14 dpw. Boxplots represent median with interquartile range; letters indicate statistically distinct groups (*p < 0.05*, pairwise Wilcox test). Images were created using fluorescent microscopy and inverting the look up tables (LUTs) uniformly in ImageJ.
